# Mitochondria as Key Players in Sarcoma Development, Progression and Drug Resistance: From Mechanistic Insights to New Treatment Opportunities

**DOI:** 10.3390/biom16101422

**Published:** 2026-10-01

**Authors:** Martina Perin, Alberto Lipani, Marco Loffa, Alberto Maria Todesco, Joanna Kopecka, Chiara Riganti

**Affiliations:** 1 Department of Oncology, University of Torino, Piazza Nizza 44/bis, 10126 Torino, Italy; martina.perin@unito.it (M.P.); alberto.lipani@edu.unito.it (A.L.); marco.loffa@edu.unito.it (M.L.); albertomaria.todesco@unito.it (A.M.T.); joanna.kopecka@unito.it (J.K.); 2 Molecular Biotechnology Center “Guido Tarone”, Piazza Nizza 44/bis, 10126 Torino, Italy

**Keywords:** sarcoma, mitochondria metabolism, mitochondria dynamics, drug resistance, immune evasion, mitochondrial-targeting drugs

## Abstract

Sarcomas are a heterogeneous group of tumors accounting for 1% and 15% of adult and child cancers, respectively. Because of their high genomic instability, biological heterogeneity and metastatic potential, sarcomas are challenging to treat. Current treatments rely on surgical resection combined with chemotherapy and radiotherapy; however, the outcome remains poor in advanced or recurrent stages. As in many other solid tumors, a growing body of evidence in sarcoma also highlights the central role of metabolic reprogramming in progression and treatment resistance. Mitochondria are key players in bioenergetic processes, redox balance and apoptotic signaling. This review will discuss the latest findings demonstrating that alterations in mitochondrial energetic metabolism, mitochondrial dynamics and mitochondria-dependent pro-apoptotic signaling promote malignant transformation, progression, immune evasion and resistance to treatments in soft-tissue and bone sarcomas. Mitochondrial pleiotropism, however, can unveil new vulnerabilities. Several compounds disrupting mitochondrial respiration, increasing the production of mitochondrial reactive oxygen species, and triggering mitochondrial-dependent apoptosis achieved promising results at a preclinical level as new anti-sarcoma agents, alone or combined with chemotherapy, radiotherapy or immunotherapy. Knowing the multifaceted roles of mitochondria in sarcoma progression and resistance may be the first step toward new treatment tools, noteworthy for future clinical development.

## 1. Introduction to Sarcomas: Classification, Clinical Management, and Prognosis

Sarcomas are a heterogeneous group of malignancies originating from bone and soft tissues and comprise more than 100 histological subtypes according to the 2020 WHO classification [1]. Their epidemiology, molecular characteristics, clinical behavior, and response to treatment vary considerably across histotypes. Osteosarcoma (OS) and Ewing sarcoma (ES) predominantly affect children and adolescents, whereas chondrosarcoma (CS) mainly occurs in adults; soft-tissue sarcomas (STSs) encompass a particularly diverse group of tumors arising from different connective tissues [2,3,4,5].

Surgical resection with adequate margins remains the standard of treatment for localized disease, frequently combined with chemotherapy and/or radiotherapy according to histology and disease stage. OS and ES are generally treated using multimodal approaches, whereas conventional CS is poorly responsive to chemotherapy and radiotherapy. Anthracycline-based chemotherapy remains the standard systemic treatment for many advanced STSs, although histology-specific and molecularly targeted treatments are increasingly available [2,3,4,5,6].

Despite these therapeutic approaches, outcomes remain poor for patients with metastatic, recurrent, or treatment-resistant disease. Prognosis differs substantially according to histological subtype, tumor grade, metastatic status and treatment response. The marked biological and clinical heterogeneity of sarcomas therefore requires specialized multidisciplinary management and complicates the development of broadly effective therapies, highlighting the need to identify subtype-specific molecular vulnerabilities and novel therapeutic strategies [2,3,4,5,6].

In several solid tumors, mitochondria mediate tumor progression and resistance. Recent evidence also depicts such a role for mitochondria in sarcomas, although this field has not been investigated in a comprehensive way. The aim of this review is to provide an overview of the mitochondrial biological processes in sarcoma progression and drug resistance. We discuss the latest evidence demonstrating how alterations in mitochondrial energetic metabolism, mitochondrial dynamics, and mitochondria-dependent signaling contribute to malignant transformation, tumor progression, immune evasion, and resistance to conventional therapies. Furthermore, we examine the emerging mitochondria-targeted therapeutic strategies, highlighting their potential as anti-sarcoma drugs or sensitizing agents capable of enhancing chemotherapy or immunotherapy efficacy. In the era of precision medicine, proposing personalized interventions targeting mitochondria may open novel and unexpected treatment options in specific subsets of sarcomas. The review was built by searching in (1) the PubMed database (https://pubmed.ncbi.nlm.nih.gov/), using the terms “sarcoma, mitochondrial metabolism, mitochondrial dynamics, mitochondria and response to chemotherapy, mitochondria and immune evasion, mitochondria and response to immunotherapy, combination treatment in sarcoma” (original works, reviews and case reports published in the period 2020–2026 (and few older references cited herein) were included, and (2) the Trials.gov database (https://clinicaltrials.gov/), selecting “sarcoma” as “condition/disease” and “mitochondria” as “other terms”; ongoing and completed intervention studies using agents targeting mitochondria were included.

## 2. Metabolic Reprogramming in Sarcomas: Biological Mechanisms and Clinical Implications

Metabolic reprogramming is a hallmark of cancer that enables malignant cells to adapt to the increased energetic and biosynthetic demands associated with uncontrolled proliferation, survival under stress conditions and metastatic dissemination. Like other malignancies, sarcomas undergo profound metabolic alterations that support tumor growth, maintenance of redox homeostasis, and resistance to therapy. Given the remarkable heterogeneity of sarcomas, metabolic adaptations may vary substantially among different sarcoma entities [7]. One of the most extensively studied metabolic features of sarcomas is the prevalence of aerobic glycolysis, commonly known as the Warburg effect. Despite the presence of oxygen, sarcoma cells preferentially metabolize glucose into lactate, a less efficient process for ATP generation but a source of intermediates for nucleotide, amino acid, and lipid biosynthesis [8]. This glycolytic phenotype is sustained by high levels of glucose transporter 1 (GLUT) and key glycolytic enzymes including hexokinase 2 (*HK2*), 6-phosphofructo-2-kinase/fructose-2,6-bisphosphatase (PFKFB family members), phosphoglycerate mutase 1 (*PGM1*), and lactate dehydrogenase A (*LDHA*), many of which are regulated by oncogenic pathways such as phosphoinositide 3-kinase/protein kinase B (PI3K/AKT), cellular myelocytomatosis (*c-MYC*), Kirsten rat sarcoma virus oncogene homolog (*KRAS*), and Yes-associated protein (YAP) signaling [7,9,10,11]. In addition, alterations in the pentose phosphate pathway and tricarboxylic acid (TCA) cycle contribute to enhanced NADPH production, biosynthetic capacity, and redox control [9], promoting tumor progression and therapy resistance.

Increasing evidence indicates that oxidative phosphorylation (OXPHOS) also plays an important role in sarcoma biology. Several sarcoma subtypes exhibit metabolic plasticity, allowing them to switch between glycolytic and mitochondrial energy production according to environmental conditions. Deregulations of mitochondrial functions and pyruvate metabolism have been implicated in this transition, supporting tumor progression but at the same time offering promising therapeutic vulnerabilities [10,12]. The heterogeneous histological subtypes make complex drawings a unique paradigm: for instance, several ncRNAs and miRNAs are known to upregulate the TCA cycle via a common mechanism, i.e., the inhibition of pyruvate dehydrogenase kinase 1 (*PDK1*) enzyme. However, the pattern of miRNAs implicated is completely different between OS and rhabdomyosarcoma (RMS) [10].

Altered amino acid metabolism constitutes another hallmark of sarcoma metabolic rewiring. Amino acids not only serve as substrates for protein synthesis but also support nucleotide production, energy generation, epigenetic regulation, and maintenance of redox balance [13]. Many sarcomas display metabolic dependencies on specific amino acids, a phenomenon known as auxotrophy. For example, low expression of argininosuccinate synthetase 1 (*ASS1*), observed in several STS subtypes, results in arginine auxotrophy and creates opportunities for therapeutic arginine depletion strategies [8,13]. Likewise, enhanced serine biosynthesis has been reported in OS and ES through upregulation of phosphoglycerate dehydrogenase (*PHGDH*), linking glucose metabolism to one-carbon metabolism and nucleotide synthesis [13]. Glutamine metabolism is also frequently deregulated, with increased expression of glutaminase (*GLS*) and glutamine transporters supporting anabolic growth, redox homeostasis, and metastatic potential. Notably, elevated *GLS1* expression has been associated with poor clinical outcomes in OS, and pharmacological inhibition of glutaminolysis has demonstrated antitumor activity in preclinical models [7,11,13].

Recent studies have further highlighted the importance of lipid metabolism in sarcoma progression. Tumor cells frequently exhibit enhanced de novo lipogenesis and fatty acid oxidation (*FAO*) to sustain membrane synthesis, signal transduction, and energy production [8]. Several enzymes involved in lipid metabolism, including ATP-citrate lyase (*ACLY*), fatty acid synthase (*FASN*), and HMG-CoA reductase (*HMGCR*), are upregulated in OS and other sarcoma subtypes, where they contribute to proliferation, migration, metastasis, and treatment resistance [11]. Moreover, dysregulation of lipid metabolic regulators such as aryl hydrocarbon receptor nuclear translocator 2 (*ARNT2*) has recently been implicated in retroperitoneal liposarcoma (*LS*), further supporting the role of lipid remodeling in sarcoma pathogenesis [14]. Recent evidence has identified mitochondrial gene signatures with prognostic value in OS [15], although several underlying mechanisms remain to be elucidated.

Collectively, these findings demonstrate that sarcomas exploit multiple interconnected metabolic pathways, including glucose, amino acid and lipid metabolism, to support tumor growth and adaptation to hostile microenvironments. Understanding the molecular mechanisms underlying metabolic reprogramming in sarcomas is not only crucial for elucidating disease biology but also for identifying novel therapeutic targets that may improve outcomes for patients with these rare and often aggressive malignancies.

### 2.1. Mitochondrial Energetic Metabolism

A critical metabolic hallmark in most sarcomas is an increased rate of TCA cycle and OXPHOS, since they provide energetic and synthetic advantages required for tumor growth, progression and survival in response to anticancer agents. However, there is not a unique behavior but rather a remarkable ability to reshape the key energetic metabolic pathways depending on the heterogeneous histotype, mutational landscape and tumor microenvironment (*TME*).

Sarcomas show high variety in chromosome numbers, not only between different histotypes but also within the same tumor. For instance, subpopulations with high or low mitochondrial respiratory capacity can be identified in STS [16]: such heterogeneity favors a rapid adaptation to the changing availability of nutrients, allowing sarcoma cells to shift from glucose to fatty acids or glutamine as preferential energetic sources. A compensatory increase in glycolysis is observed in sarcoma cells that lose mitochondrial DNA because of their intrinsic genetic instability, generating intratumor clones characterized by a glycolytic-sustained epithelial-to-mesenchymal transition [17].

Changes in TCA cycle activity have been described in different sarcoma subtypes. OS shifts from a TCA-dependent phenotype to a glycolytic-dependent phenotype in response to chemotherapy. This phenotype is driven by transforming growth factor-β (*TGFβ*) that suppresses succinate dehydrogenase (*SDH*) activity in the TCA cycle and stabilizes hypoxia-inducible factor-1α (*HIF1α*), upregulating glycolytic genes. Concurrently, TGFβ supplementation enhances resistance to methotrexate and cisplatin both in vitro and in vivo, while pharmacological inhibition of HIF1α restores chemosensitivity [18]. In gastrointestinal stromal tumors (*GIST*), inactivating mutations in SDH subunits (A–D) are well documented. The genetic loss of SDH determines the accumulation of succinate, the inhibition of α-ketoglutarate-dependent dioxygenases and the activation of HIF1α-dependent pathways as in OS. However, GIST cells simultaneously increase TCA cycle anaplerotic pathways and glycolysis to meet the energetic demands of cancer cells [17,19], rather than shifting from the TCA cycle to glycolysis. In CS, the most frequent alteration affecting the TCA cycle is the presence of oncogenic variants of isocitrate dehydrogenase 1 (*IDH1*) that results in the abnormal production of the oncometabolite 2-hydroxyglutarate [20], promoting tumor growth. Conversely, high OXPHOS activity is also a hallmark of most sarcoma subtypes, except undifferentiated pleomorphic sarcomas [16]. The increased activity of the electron transport chain (*ETC*) complexes influences sarcoma growth and sensitivity to therapies in different sarcoma subtypes. For instance, the oncogenic E76K mutation in Src homology region 2 domain-containing phosphatase-2 (*SHP2*) promotes sarcomagenesis by increasing OXPHOS, production of mitochondrial ATP and reactive oxygen species (ROS), and mitochondrial metabolism of glucose and amino acids. These events drive the malignant transformation of mesenchymal stem cells (*MSC*) into sarcoma stem-like cells. Importantly, pharmacological inhibition of SHP2 signaling or ETC complexes prevents this transformation [21], highlighting the role of ETC in tumor initiation and stemness maintenance. Beyond tumor onset, sarcoma progression also relies on increased OXPHOS. Via a mitochondrial proteomic analysis, increased assembly and activity of cytochrome c oxidase (complex IV) has been documented in fibrosarcoma (FS). In particular, two complex IV subunits, cytochrome c oxidase subunit 2/3 (MT-CO3) and cytochrome c oxidase (COX6A1), are upregulated in FS samples and associated with enhanced respiratory activity and oxygen consumption. Enzymes involved in FAO such as carnitine palmitoyltransferase 1 (*CPT1*) and acyl-CoA thioesterase 9 (*ACOT9*) are significantly upregulated as well [22], likely promoting the FAO → TCA cycle → OXPHOS metabolic flux. Differently from FS, in OS, complex I drives progression and metastatization more than complex IV; a high mutation burden in the mitochondrial encoded protein NADH dehydrogenase subunit 1 (*MT-ND1*), which is necessary for the electron transfer within complex I, reduces OS cell growth and dissemination [23]. Given the intrinsic instability of genomic and mitochondrial DNA within OS, there may be heterogeneous scenarios, depending on the amount of mutations accumulated within the *MT-ND1* gene; only above a specific threshold, mutations compromise OXPHOS efficiency and act as endogenous tumor brakes [23]. Another key regulator of mitochondrial metabolism in OXPHOS is methyltransferase-like 17 (*METTL17*), which is significantly overexpressed in OS tissues and cell lines compared with healthy osteoblasts. Its silencing reduces the expression of several ETC proteins, including MT-CO2, SDHB, Ubiquinol-cytochrome c reductase binding protein (*UQCRB*) and NADH-ubiquinone oxidoreductase subunits, leading to impaired OXPHOS activity and ATP production. Both genetic silencing and pharmacological inhibition of *METTL17* significantly reduce osteosarcoma growth in xenograft models thanks to the impairment of mitochondrial metabolism [24].

Also, RMS relies on OXPHOS, but the metabolic driver seems different. Indeed, Adenine Nucleotide Translocator 1 (*ANT1*) has been identified as a key regulator of increased OXPHOS in this sarcoma histotype [25]. Transcriptomic analyses have revealed that ANT1 is significantly lower in RMS, reaching a 24-fold reduction compared to healthy skeletal muscles. This downregulation induces a metabolic switch conferring a selective advantage that sustains cell proliferation and confers resistance to stress-induced death. Cells with low ANT1 levels display increased basal oxygen consumption rate (*OCR*) and enhanced spare respiratory capacity (*SRC*), coupled with decreased basal extracellular acidification rate (*ECAR*). The downregulation of ANT1 shifts RMS cells towards an oxidative state similar to regenerating myogenic precursors, essential for maintaining the tumor’s highly proliferative phenotype [25]. With a slight difference from RMS, in OS the ANT3 isoform is predominant in promoting mitochondrial fitness, cell survival and chemoresistance by interacting with EF-hand domain-containing protein 1 (*EFHD1*). The *EFHD1-ANT3* axis rewires OS metabolism, increasing ATP production and respiratory capacity and reducing ROS levels. The therapeutic relevance of this mechanism is confirmed by the ANT3 inhibitor carboxyatractyloside that induces mitochondrial permeability transition pore (mPTP) opening and restores cisplatin sensitivity both in vitro and in vivo [26].

Methylthioadenosine phosphorylase (*MTAP*), a key enzyme involved in the methionine and adenosine salvage pathway, fueling the TCA → OXPHOS metabolic flux [27], represents another metabolic vulnerability that can be exploited therapeutically. *MTAP* is often deleted in OS [28]. Methionine deprivation drastically reduces cell growth and induces apoptosis in vitro and in patient-derived xenografts, because of the impaired TCA cycle and OXPHOS. Mechanistically, low methionine levels and *MTAP* activity reduce the activation of C1orf112, a protein preserving DNA integrity and chromatin remodeling, and specifically increasing the transcription of mitochondrial genes involved in OXPHOS, ATP production and ROS buffering in OS [29]. Beyond its role in tumor progression, an active OXPHOS can promote chemoresistance. Human doxorubicin-resistant OS cells have high OXPHOS levels, associated with increased mitochondrial biogenesis, metabolic flux through the TCA cycle, FAO and OXPHOS. This metabolic phenotype leads to high mitochondrial ATP levels that boost the catalytic activity of drug efflux transporters of the ATP binding cassette (*ABC*) family, such as P-glycoprotein (*P-gp*), which is indeed increased in chemoresistant OS. This metabolic phenotype can be exploited using a mitochondrial-targeted doxorubicin (*mtDox*), which increases oxidative stress within mitochondria, impairs OXPHOS and bypasses P-gp-mediated efflux [30]. Differently from OS, in ES the loss of genes required for complexes III and IV (but not I, II or V) confers resistance to the lysine-specific demethylase-1 (*LSD1*) inhibitors (SP-2509). Such mitochondrial deficiency induces a transcriptional state resembling LSD1 inhibition, thereby reducing drug efficacy. These findings highlight a previously unrecognized connection between mitochondrial function and epigenetic therapy response in ES [31]. Another regulator of OXPHOS and chemoresistance in OS is the nuclear receptor RAR-related orphan receptor γ (*RORγ*), whose knockdown or pharmacological inhibition reduces cell growth and induces apoptosis. *RORγ* inhibition reduces FAO, increases glycolytic activity, and regulates peroxisome proliferator-activated receptor gamma coactivator 1β (*PGC1β*), a transcriptional coactivator controlling mitochondrial biogenesis and oxidative metabolism. Importantly, *RORγ* inhibition suppresses tumor growth in methotrexate- and cisplatin-resistant patient-derived xenograft models and reduces the expression of drug resistance-associated transporters. High levels of *RORγ* and *PGC1β* are associated with poor prognosis, highlighting the *RORγ–PGC1β* axis as a key regulator of mitochondrial metabolism and a potential therapeutic target in OS [32].

Overall, the examples highlight a remarkable metabolic plasticity of sarcoma cells depending on the sarcoma histotype, heterogenous intratumor genotype and extracellular signals. Sarcoma cells show the remarkable ability of tuning their metabolism toward the best adaptive mechanism, granting survival or escape from the cytotoxic damage elicited by chemotherapy.

### 2.2. Mitochondrial Importers and Transporters

To sustain the high metabolic demands required for tumor growth and survival, sarcomas frequently increase mitochondrial import and transport systems. These proteins, localized on both the outer and inner mitochondrial membranes, play critical roles in mitochondrial metabolism, redox homeostasis and resistance to cell death.

Among them, translocase of the outer mitochondrial membrane 20 (*TOMM*) has emerged as an important regulator of sarcoma metabolism. *TOMM20* is overexpressed in high-grade CS and is associated with increased *OXPHOS*. Functional studies demonstrate that *TOMM20* promotes mitochondrial metabolism, cell proliferation, and resistance to doxorubicin, gemcitabine and palbociclib, ultimately enhancing tumor growth [33]. More recently, similar findings have been reported in FS, where *TOMM20* overexpression enhances *OXPHOS*, promotes metabolic flexibility using glutamine and lactate instead of glucose and maintains redox homeostasis by increasing antioxidant defenses. These adaptations support tumor growth, invasiveness and drug resistance, whereas TOMM20 depletion impairs mitochondrial metabolism, increases oxidative stress, and suppresses tumor progression [34]. Strikingly, *TOMM20* is a potential therapeutic target in both CS and FS: if its role is confirmed also in other sarcomas histotypes, such as OS and ES, it may be noteworthy of being further investigated as a potential therapeutic target, present across multiple sarcoma subtypes.

Components of the translocase of the inner mitochondrial membrane (*TIMM*) complex and solute carrier (*SLC*) proteins have also emerged as oncogenic drivers in OS. *TIMM13* is significantly upregulated in OS and correlates with poor patient prognosis [35]. While *TIMM13* promotes mitochondrial integrity and cellular fitness, its depletion induces oxidative stress, impairs mitochondrial function, and suppresses proliferation, migration, and tumor growth. Similarly, *TIMM50* has been identified as a tumor promoter in *RMS*, where it is inhibited by the tumor-suppressive microRNA miR-7 [36]. MiR-7 overexpression downregulates *TIMM50* and other mitochondrial genes, resulting in mitochondrial dysfunction, loss of membrane potential, increased oxidative stress, and reduced tumor growth [36]. Another mitochondrial transporter recently implicated in OS progression is *SLC25A10*. Elevated *SLC25A10* expression is associated with poor prognosis and metastatic disease, while functional studies demonstrate that *SLC25A10* promotes proliferation, migration, and epithelial–mesenchymal transition [37]. *SLC25A10* supports mitochondrial function by maintaining *OXPHOS* and ATP production, preserving membrane potential and redox balance. Redox homeostasis is a critical determinant of sarcoma progression and therapeutic response. While moderate ROS levels can promote tumor growth by activating proliferative pathways, inhibiting apoptosis, and remodeling the tumor microenvironment, excessive oxidative stress is detrimental to cancer cells and therefore must be tightly controlled [38]. This vulnerability has attracted considerable therapeutic interest toward those mitochondrial transporters upregulated in OS and necessary to limit mitochondrial ROS increase.

### 2.3. Changes in Redox Homeostasis

The heterogeneous levels of ROS produced within sarcoma cells can be due to the different intratumor activity of *OXPHOS* and mitochondrial importers, but also to the differential expression and activity of specific proteins controlling redox balance (Figure 1). As it occurs for other mitochondria-related processes, high differences between sarcoma histotype and within the same tumor have also been reported for redox homeostasis.

In OS, the mitochondrial oxaloacetate decarboxylase Fumarylacetoacetate Hydrolase Domain-Containing 1 (*FAHD1*), which promotes TCA cycle activity and mitochondrial ROS production, is often downregulated: the decrease in mitochondrial ROS below the toxic threshold favors cell proliferation. Conversely, its overexpression increases mitochondrial oxidative damage, by shutting down mitochondrial metabolism and favoring a metabolic shift toward glycolysis [38]. The mitochondrial APE1 (mtAPE1), which repairs mitochondrial DNA lesions, also protects OS cells from cisplatin-induced damage by limiting the damage elicited by ROS of mitochondrial origin [39]. Similarly to mtAPE1, the deubiquitinase OTULIN also mediates cisplatin resistance by stabilizing glutathione peroxidase 4 (*GPX4*), which in turn limits ROS accumulation and mitochondrial damage-triggered apoptosis in OS models [32].

If, in OS, high levels of ROS are deleterious and prevent cell proliferation, a completely different scenario occurs in ES, whose cells continuously deal with constitutively high levels of ROS. Being protected by high levels of antioxidant enzymes lacking in OS, CS cells are stimulated to proliferate by ROS. Indeed, in ES the Ewing sarcoma breakpoint region 1–Friend leukemia integration 1 (*EWSR1–FLI1*) fusion oncogene induces SRY-box transcription factor 6 (*SOX6*), which in turn upregulates the thioredoxin-interacting protein (*TXNIP*), a negative regulator of the antioxidant thioredoxin reductase (*TrxR*) enzyme. This cascade produces a controlled level of ROS that promotes tumor growth. Disrupting this balance, e.g., by increasing mitochondria ROS with Elesclomol, is instead an effective anti-cancer and chemosensitizing strategy in ES with high expression of *SOX6* [40]. These findings highlight once again that different sarcoma subtypes show heterogenous behavior toward a common biological event, such as mitochondrial ROS production and redox homeostasis.

### 2.4. Mitochondrial Dynamics and Mitophagy

Mitochondrial fusion and fission are essential processes regulating mitochondrial homeostasis and metabolism, and their dysregulation is increasingly recognized as a hallmark of cancer. In OS, mitochondrial dynamics contribute to tumor progression and chemoresistance by modulating mitochondrial morphology and function (Figure 2). This is particularly evident in OS stem cells, characterized by higher interconnected mitochondrial network and higher resistance to cisplatin and doxorubicin compared with non-stem tumor cells. This phenotype is associated with sustained ATP production, low ROS accumulation, and high expression of the fission regulator dynamin-related protein 1 (DRP1). Importantly, DRP1 depletion restores chemosensitivity by increasing mitochondrial-derived oxidative stress [41].

The role of DRP1, however, is controversial, depending on sarcoma histotypes. In RMS, DRP1 loss is not sufficient to impair sarcoma growth or overcome drug resistance, suggesting the existence of compensatory, DRP1-independent fission mechanisms. Indeed, alternative fission adaptors such as mitochondrial fission factor (*MFF*) and fission 1 protein (*FIS1*) are upregulated in DRP1-depleted RMS, where they play a predominant role in promoting tumor progression. Consistently, the prognostic significance of DRP1 is subtype-specific, being associated with poor prognosis in OS and favorable outcomes in RMS [42].

Mitochondrial dynamics also influence the osteogenic differentiation of OS cells, where an early fission phase is followed by mitochondrial fusion and network formation during later stages of differentiation [43], highlighting another aspect controlled by mitochondria dynamics.

Mitophagy, the selective autophagic degradation of mitochondria, represents a critical mechanism balancing cell survival and apoptosis. In cancer, dysregulated mitophagy can promote cell survival and therapy resistance by limiting mitochondrial-dependent apoptotic signaling [44]. This process is mainly controlled through ubiquitin-dependent and ubiquitin-independent pathways. In the canonical ubiquitin-dependent pathway, PTEN-induced kinase 1 (*PINK1*) accumulated on damaged mitochondria activates PARKIN RBR E3 ubiquitin protein ligase (PARKIN), triggering ubiquitination cascades that recruit LC3-positive autophagosomes. Alternatively, receptor-mediated pathways involving BCL2 interacting protein 3 (*BNIP3*), *BNIP3* like/NIP-3 like protein X (*BNIP3L*/*NIX*), and FUN14 domain-containing 1 (*FUNDC1*) directly interact with LC3 and GABA type A receptor-associated protein (*GABARAP*) proteins to initiate mitophagy [45,46]. Mitophagy-related gene signatures—including autophagy-related 4D (*ATG4D*), ribosomal protein S27A (*RPS27A*) and TOMM20 genes as well as kallikrein-related peptidase 2 (*KLK2*), neurexin 1 (*NRXN1*), hes family bHLH transcription factor 5 (*HES5*), olfactory receptor family 2 subfamily W member 3 (*OR2W3*), and heparan sulfate-glucosamine 3-sulfotransferase 4 (*HS3ST4*)—are associated with poor clinical outcomes, altered immune infiltration patterns, and low response to chemotherapy or immunotherapy [47,48]. Intratumor heterogeneous mitophagy, associated with distinct tumor niches, characterizes OS: indeed, high-mitophagic cells have enhanced interactions with endothelial cells, activation of angiogenic pathways and metastatic phenotype, whereas low-mitophagic cells preferentially interact with immune and stromal populations [49]. Beyond metastatization, mitophagy mediates low response to treatment and poor clinical outcome in OS [46,48]. As for many other biological processes related to mitochondria, the role of mitophagy in tumor progression and drug response is highly context-dependent across sarcoma subtypes. Differently from OS, in dedifferentiated LS, mitophagy is associated with tumor-suppressive functions. In this setting, tumor protein p53-inducible nuclear protein 2 (*TP53INP2*) promotes the PARKIN-dependent mitophagic degradation of the oncogenic factor YAP. Accordingly, low levels of *TP53INP2* are associated with disease progression and poor prognosis, indicating that loss of mitophagy-mediated YAP degradation contributes to LS progression [50]. These findings highlight a dual role of mitophagy in sarcoma biology, acting either as a mechanism of tumor adaptation or as a tumor-suppressive pathway, depending on sarcoma genetics and histotype.

Another recently studied process dependent on mitochondria and controlling cell death is cuproptosis, a copper-induced form of regulated cell death occurring when copper ions accumulate within mitochondria and bind lipoylated proteins of the TCA cycle, causing protein aggregation, mitochondrial proteotoxic stress, and ultimately cell death through a process dependent on ferredoxin 1 (*FDX1*) [51]. High expression of the cuproptosis-related genes *FDX1*, *TOMM20*, *NADH*:ubiquinone oxidoreductase subunit B9 (*NDUFB9*), *COX*, mitofusin 2 (*MFN2*), and ATPase H^+^transporting V1 subunit E1 (*ATP6V1E1*) has been found to be associated with clinical positive outcome and enrichment of specific immune populations in OS. This is linked with positive response to immunotherapy. Conversely, the association is not univocal with response to chemotherapeutic drugs or targeted therapies, where each gene is an independent negative or positive factor [51]. This diverse scenario indicates that cuproptosis-related genes also have a highly context-dependent meaning.

Each enzyme related to mitochondria may have pro- or anti-tumor effects depending on the expression and activity of the other. This is part of the intrinsic heterogeneity in genetic and metabolic phenotypes of sarcomas. At the same time, the works discussed indicate that a plethora of mitochondrial-related biological processes, including energetic and redox metabolism, fusion/fission balance, mitophagy, and cuproptotic cell death, are prognostic biomarkers, mechanistic drivers of sarcoma progression and response to treatment. Of note for the scope of this review, specific alterations of mitochondria metabolism and functions support resistance to chemotherapy in different types of sarcomas, as summarized in Table 1.

These findings led to investigating whether specific mitochondrial-dependent pathways can be exploited as therapeutic targets. In interpreting these therapeutic vulnerabilities, it is important to distinguish between direct mitochondrial targets and indirect modulators of mitochondrial function. Direct mitochondrial targeting requires evidence of physical accumulation within mitochondria or engagement of a defined mitochondrial component, such as ETC complexes, mitochondrial transporters, mitoribosomes, or mitochondrial membrane proteins. Conversely, interventions acting on upstream signaling, cellular metabolism, autophagy, redox balance, or TME may ultimately alter OXPHOS, ROS production, mitochondrial dynamics, or apoptosis without engaging mitochondria as a primary molecular target. Accordingly, mitochondrial dysfunctions alone should not be considered sufficient evidence of direct mitochondrial targeting.

## 3. Pharmacological Strategies Targeting Mitochondria in Sarcoma

For the purpose of this review, the pharmacological approaches discussed below—repurposed drugs, synthetic or natural compounds, nanoformulations (Figure 3)—are classified into three conceptual categories: (i) direct mitochondria-targeted agents, for which mitochondrial localization or engagement of a defined mitochondrial molecular target has been demonstrated; (ii) indirect mitochondria-targeted agents, which inhibit a defined mitochondrial process, such as OXPHOS or mitochondrial translation, although selective mitochondrial accumulation has not necessarily been established; (iii) mitochondrial upstream controllers, which primarily act through cell signaling, redox regulation, autophagy, metabolic adaptations or TME changes, producing—as downstream consequences—mitochondrial dysfunctions or mitochondria-dependent cell death. This distinction is particularly important because mitochondrial ROS accumulation, membrane depolarization, ATP depletion, and cytochrome c release may accompany general cellular toxicity and do not, by themselves, demonstrate that mitochondria are the primary pharmacological target.

### 3.1. Drug-Repurposing Approaches

The growing understanding of mitochondrial metabolism in sarcoma has stimulated interest in drug repurposing as a strategy to identify novel therapeutic applications for compounds already approved for clinical use. Compared with de novo drug development, repurposing offers several advantages, including established pharmacokinetic and safety profiles, lower development costs, and faster clinical translation. Increasing evidence indicates that a variety of approved drugs exert antitumor activity by altering mitochondrial metabolism, redox homeostasis, and mitochondria-dependent cell death pathways. However, for several of these agents, mitochondria appear to be downstream effectors rather than the primary molecular target.

Among repurposed compounds that indirectly perturb mitochondrial metabolism, vitamins have attracted considerable attention because of their relatively low cost, safety and deep understanding of pharmacokinetics and pharmacodynamic profile. High-dose vitamin C (ascorbate) has demonstrated selective cytotoxicity against OS cells through a pro-oxidant mechanism: by generating hydrogen peroxide through iron-dependent reactions, ascorbate induces intracellular and mitochondrial ROS accumulation, calcium dysregulation, and mitochondrial dysfunction. These effects result in mitochondrial membrane loss, impaired OXPHOS, decreased ATP production, and cell death. The available evidence supports mitochondrial dysfunction as a downstream component of ascorbate-induced oxidative toxicity, rather than demonstrating direct engagement of a mitochondrial molecular target. Importantly, vitamin C also reduces tumor growth in OS xenograft models, supporting its potential as an adjunct therapeutic strategy [52]. Vitamin C is by far the nearest vitamin to clinical application, since it has been tested in clinical trials. On the other hand, given the multifaceted role that ROS plays in different sarcomas—being cytotoxic in OS and pro-tumoral in ES—its application may be limited specifically to OS.

Another vitamin whose anticancer activity involves mitochondria as a downstream target is calcitriol or 1α,25-dihydroxyvitamin D3. In RMS cells, calcitriol induces cell-cycle arrest and mitochondria-dependent apoptosis accompanied by disruption of mitochondrial architecture [53]. Since calcitriol primarily acts through a vitamin D receptor-dependent transcriptional program, mitochondria are likely indirect targets. Although not yet tested in clinical trials, calcitriol also looks promising considering the strong physiological effect that vitamin D3 has on bones.

Several antipsychotic drugs have also demonstrated anticancer activity accompanied by mitochondrial alterations. Clozapine has been shown to disrupt redox homeostasis in LS cells by increasing mitochondrial oxidative stress and inducing mitochondrial fragmentation, ultimately leading to cell death [54]. Similarly, trifluoperazine (TFP) inhibits the growth of OS xenograft models through mitochondrial ROS accumulation and loss of mitochondrial membrane potential. Mechanistically, TFP suppresses AKT signaling, leading to *TXNIP* upregulation, mitochondrial dysfunction, and activation of apoptotic pathways. This sequence suggests that the inhibition of AKT/*TXNIP* signaling precedes mitochondrial dysfunction [55]. Although antipsychotic drugs are interesting because they are effective in at least two sarcoma histotypes, the main limitation is their concomitant effects on the central nervous system. Without selective delivery to tumors or the definition of therapeutic windows characterized by antitumor effects without psychotropic effects, the evidence on clozapine and TFP risks being confined to preclinical development. Moreover, because mitochondrial fragmentation, ROS accumulation, and membrane depolarization may also occur during nonspecific cellular injury, additional target-engagement studies are required to establish whether mitochondria are primary pharmacological targets of these antipsychotic agents.

Among currently approved drugs, metformin represents one of the most attractive candidates for sarcoma therapy. Beyond its established role in type 2 diabetes, metformin directly inhibits mitochondrial complex I and induces profound metabolic reprogramming. In OS stem cells, metformin disrupts mitochondrial integrity, increases ROS production and decreases ATP synthesis, activating mitochondria-dependent apoptosis. In vivo, these effects slow down tumor growth and metastatic progression [56]. Metformin may also exert synergistic effects when combined with agents that perturb mitochondrial function through complementary mechanisms. For instance, the combination of metformin and nitroglycerin significantly reduced FS growth in vivo because of the simultaneous impairment of mitochondrial bioenergetics and HIF-1α-dependent signaling, which resulted in reduced tumor vascularization and increased apoptosis [57]. Also in ES stem cells, metformin impairs ATP production and mitochondrial membrane potential, triggering apoptosis and overcoming doxorubicin resistance in vitro [58]. The effects of metformin on multiple sarcoma subtypes, together with its well-known pharmacokinetic and pharmacodynamic profile, make this drug noteworthy for further development in clinical settings.

Additional repurposed compounds activate mitochondria-dependent apoptosis, acting downstream of perturbed mitochondria. Disulfiram combined with copper (DSF-Cu), originally developed for the treatment of alcohol dependence, induces mitochondrial depolarization, ROS accumulation and cytochrome c release in OS cells, activating mitochondria-dependent apoptotic pathways and suppressing tumor growth [47]. Mitochondrial depolarization and cytochrome c release should be interpreted as components of ROS-mediated cell death rather than evidence of direct mitochondrial engagement. Similarly, local anesthetics such as lidocaine and ropivacaine have recently been shown to impair mitochondrial function in OS cells. Both agents promote mitochondrial fragmentation, membrane depolarization, and ROS accumulation, leading to increased apoptosis and reduced proliferation and migration [59]. Like disulfiram, the mitochondrial alterations induced by local anesthetics may represent downstream consequences of a broader cellular toxicity, and their mitochondrial specificity remains to be demonstrated. The main issue to be solved for disulfiram and anesthetics is achieving specificity without exerting side effects. Finding a suitable therapeutic window is still an open challenge.

### 3.2. Synthetic Compounds and Metal-Based Drugs Targeting Mitochondria

Based on the metabolic dependence on OXPHOS of most sarcoma subtypes, agents targeting ETC components, accumulating within mitochondria or indirectly altering energetic metabolism have been synthesized.

MS-L6, a dual complex I inhibitor and mitochondrial uncoupler, reduces ATP production, induces metabolic rewiring toward glycolysis, and exerts both cytostatic and cytotoxic effects in RMS, while showing limited systemic toxicity in vivo [60]. Another complex I inhibitor, IACS-010759, has shown increased production of mitochondrial ROS that overcome DNA repairing systems and synergize with radiotherapy in FS animal models [61]. These agents can be classified as direct mitochondria-targeting drugs because they inhibit a defined mitochondrial respiratory complex. Nevertheless, evidence of complex I inhibition does not necessarily demonstrate preferential accumulation in sarcoma mitochondria, and their effects on OXPHOS-dependent healthy tissues must also be considered.

Mitochondrial respiratory complexes can also be directly targeted through destabilization of iron–sulfur (Fe–S) cluster-containing proteins. In ES the compound SP-2509 destabilizes Fe–S-dependent components of complexes I and III, reducing respiratory capacity and inducing antitumor activity independently of its originally proposed LSD1 inhibitory function [62,63], displaying a novel therapeutic vulnerability for this aggressive sarcoma. Metal-based compounds have also emerged as attractive mitochondrial-targeting agents. Palladium complexes exhibit greater cytotoxicity than cisplatin in OS models by inducing lysosomal and mitochondrial depolarization, cytochrome c release and apoptosis, suppressing clonogenic growth [64]. Because these compounds simultaneously induce lysosomal and mitochondrial depolarization, the available evidence does not establish mitochondria as their exclusive or primary target and remains compatible with broader organelle or cellular toxicity. Among the compounds directly targeting mitochondria, photoactivatable iridium complexes have emerged as effective agents against RMS cells, particularly within cancer stem cell-enriched populations. Upon light activation, these compounds accumulate in mitochondria, induce ROS production, promote mitochondrial permeability transition, and trigger necrotic cell death [64], highlighting their potential to target apoptosis-resistant tumor cells. Phenethyl isothiocyanate (PEITC) also induces mitochondrial ROS accumulation, loss of respiratory chain proteins, ATP depletion, and mitochondrial depolarization, ultimately triggering apoptosis, ferroptosis, and autophagy in OS [65].

Another strategy of altering mitochondrial function involves inhibition of mitochondrial protein synthesis. Tigecycline, an FDA-approved antibiotic with high affinity for mitochondrial ribosomes, selectively targets OS cells characterized by elevated mitochondrial biogenesis and inhibits the translation of mitochondrially encoded respiratory chain proteins, suppressing OXPHOS and significantly impairing tumor growth [66]. A similar approach is represented by demethylzeylasteral (DEM), which inhibits *METTL17*, a regulator of mitochondrial RNA methylation and mitoribosome assembly. DEM causes widespread depletion of respiratory chain proteins, resulting in impaired OXPHOS and ATP production, mitochondrial depolarization, and cytotoxicity in OS cells [24]. Tigecycline and DEM can be classified as indirect mitochondria-targeted agents because they interfere with defined components of mitochondrial protein synthesis and mitoribosome assembly, respectively. However, selective accumulation within sarcoma mitochondria has not been demonstrated, and the possibility of additional cellular effects on general translation should be considered.

Collectively, these studies identify different levels of mitochondrial engagement. Direct or indirect mitochondria-targeting mechanisms are elicited by inhibitors of defined ETC components, mitochondrial Fe–S proteins, or mitochondrial translation, and for compounds whose mitochondrial accumulation has been demonstrated. By contrast, for several metal-based or redox-active compounds, mitochondrial depolarization, ROS production, and mitochondria-dependent apoptosis are the downstream consequences of broader cellular toxicity. The clinical translation of synthetic compounds is limited by at least three factors: (i) the poor specificity of targeting sarcoma mitochondria and the risk of inducing systemic toxicity; (ii) the low level of information about their pharmacokinetics and drug–drug interactions that must be obtained with ad hoc studies in animal models; (iii) the high plasticity and heterogeneity of mitochondria in sarcoma cells. Since sarcoma cells are quick to shift their energetic metabolism from OXPHOS to glycolysis upon the pressure of mitochondrial-targeting agents [17], this adaptability can lower the production of ROS by mitochondria while preserving an adequate ATP, limiting the efficacy of compounds directed toward ETC complexes. Moreover, within the tumor bulk, specific clones with low OXPHOS activity may emerge, generating persister populations poorly responsive to mitochondrial-targeting drugs Notwithstanding the promising results at preclinical levels, several limitations must be addressed before proposing these compounds in clinical settings.

### 3.3. Natural Products Targeting Mitochondrial Metabolism

Beyond approved drugs and synthetic products, natural compounds have emerged as another important source of agents capable of perturbing mitochondrial function. For most of these compounds, however, mitochondrial ROS production, permeability transition, or apoptosis have been demonstrated without the identification of a primary mitochondrial molecular target. For instance, modified lignin derivatives deplete OS cells of intracellular glutathione, leading to ROS accumulation and inducing mitochondrial permeability transition pore opening, cytochrome c release, and activation of apoptosis [67]. Because glutathione depletion precedes mitochondrial injury, these findings support an indirect redox-mediated mechanism rather than direct mitochondrial engagement. Similarly, Metarhizin C, a fungal metabolite isolated from *Tolypocladium album*, selectively inhibits mitochondrial OXPHOS and displays enhanced cytotoxicity under glucose-deprived conditions, when the energetic needs of OS cells strictly depend on OXPHOS [68]. The direct mitochondrial target and mitochondrial localization of Metarhizin C remain to be defined.

Other natural products induce mitochondria-dependent apoptosis through specific molecular circuits without directly targeting mitochondrial proteins. The plant-derived product corynoline, extracted from *Corydalis incisa*, activates the Src/c-Jun N-terminal kinase (JNK) pathway, resulting in mitochondrial damage, loss of membrane potential, activation of apoptotic cascades, and suppression of OS growth both in vitro and in vivo [69]. Likewise, the flavonoid cirsiliol inhibits the PI3K/AKT pathway, promotes Forkhead box protein O1 (*FOXO1*) activation, and induces both mitochondrial-dependent apoptosis and autophagy, resulting in sensitization to methotrexate in resistant OS xenografts [70]. Similar pro-apoptotic effects have been reported for breviscapine, another flavonoid that increases *Bax*, cytochrome c release, and caspase activation while reducing tumor growth without significant toxicity [71]. These act as upstream mitochondrial modulators, because Src/JNK or PI3K/AKT/*FOXO1* signaling appears to be affected upstream of mitochondrial damage and apoptosis.

Beyond apoptosis, several natural compounds exploit ferroptosis-related vulnerabilities. Shikonin induces mitochondrial ROS accumulation and activates the HIF-1α/hemeoygenase-1 (HO-1) axis, resulting in ferroptotic cell death in OS [72]. Similarly, *Marsdenia tenacissima* extracts promote iron accumulation, oxidative stress, *PINK1*/Parkin-dependent mitophagy, and ferroptosis, leading to significant inhibition of tumor growth in vivo [73]. In both cases, mitochondrial ROS production and mitophagy cause cell death, indicating that the engagement of mitochondria is indirect.

Interestingly, preclinical studies in animal models show that most natural compounds have anti-sarcoma activity when used alone. This feature is important when dealing with sarcomas refractory to the standard treatments, because finding new effective drugs is compelling. However, natural products must also meet specific requirements before being prioritized for consideration in clinical settings. First, the purity of natural extracts must be carefully evaluated. Second, although safe at the concentrations used in animal models, most natural compounds are metabolized by cytochrome P450 systems: the spectra of metabolites produced, as well as their activating or inhibiting effects on cytochromes, must be carefully evaluated before conceiving studies in human subjects, because unexpected toxicities or drug–drug interactions cannot be excluded.

### 3.4. Nanoformulations Targeting Mitochondria in Osteosarcoma

Since one of the main limitations in using agents targeting mitochondrial metabolism is the poor specificity toward tumors, an increasing number of nanomedicine-based tools have been designed to overcome this limitation, with the aim of increasing the ratio between antitumor benefits and healthy tissue damage. Cargo carried by nanoparticles includes inhibitors of energetic metabolism, inducers of mitochondrial ROS or agents interfering with mitochondrial dynamics.

One example is the OPDEA-PDCA formulation, which consists of a stable mitochondria-directed polymer micelle (OPDEA) conjugated with dichloroacetate (DCA), a specific inhibitor of *PDK1* [74], which is highly expressed in OS, associated with immune escape and low survival [75]. *PDK1* inhibition enhances OXPHOS in OS cells, promoting mitochondrial oxidative stress and mPTP opening, triggering pyroptotic cell death associated with immunogenic cell death. At the same time, dying OS cells release soluble Programmed Death-Ligand 1 (PD-L1), relieving T cell exhaustion. OPDEA-PDCA-treated xenografts have smaller tumors with necrotic areas and higher Interferon gamma (*INFɣ*) expression [74], highlighting that metabolic rewiring can simultaneously induce cancer cell death and stimulate anti-tumor immunity.

The induction of immune response following the production of mitochondrial ROS upon OXPHOS alterations is the principle exploited by pH-sensitive nanocages simultaneously releasing Ca^++^, Elesclomol and Cu_2_O. Elesclomol allows mitochondrial-specific delivery [76]; Ca^++^ accumulation forces induction of the TCA cycle and OXPHOS, increasing the production of mitochondrial ROS, whose generation is amplified by Cu ions [77]. Such mitochondrial dysfunction is associated with the release of damage-associated molecular patterns (DAMP) including calreticulin, ATP and high-mobility group box 1 (*HMGB1*) that trigger immunogenic cell death followed by increased activation of cytotoxic CD8^+^ T lymphocytes and M1 macrophages [77]. The same strategy to induce cell death by increasing mitochondrial ROS has been exploited by using mesoporous silica nanoparticles releasing metformin, which impairs mitochondria OXPHOS, and Prussian Blue, which acts as nanozyme with peroxidase-like activity, catalyzing the conversion of hydrogen peroxide into hydroxyl radicals [78].

An innovative approach combining selective mitochondrial localization and mitochondrial dynamics alteration is the nanoformulation QCAi-Cy7d, which is composed of the proto-oncogene serine/threonine-protein kinase (*PIM1*) inhibitor QCAi and the cyanine dye Cy7d that acts as a selective mitochondrial vector. Molecular docking analyses demonstrated that the cargo of this nanoformulation is released within the mitochondria, where the inhibition of *PIM1* interferes with the activity of DRP1; the low mitochondrial fission increases intratumor apoptosis in OS xenografts [79]. This formulation meets the criteria for direct mitochondrial targeting, showing both intramitochondrial delivery and engagement of a mitochondrial molecular pattern. Another selective vector to mitochondria is triphenylphosphonium (TPP) that has been conjugated with self-assembled poly(oligo ethylene glycol methyl ether methacrylate) nanoparticles loaded with the anticancer drug 4-(N-(S-penicillaminylacetyl)amino)phenylarsenonous acid; these carriers have shown good antitumor efficacy against synovial sarcoma cells in vitro, thanks to preferential uptake and delivery of the cargo directly to mitochondria [80]. Since OS is the most common sarcoma type, the vast majority of nanoformulations have been developed against this tumor. The strategies of targeting mitochondria by altering energetic and redox metabolism, or mitochondria dynamics, can be translated to other sarcoma subtypes where mitochondria functions are critical for cell survival.

Although tested simply at the preclinical levels, nanoformulations offer the advantages of being more selective for tumor tissues, for passive targeting due to enhanced permeability and retention effect, or for active targeting. Second, they can simultaneously deliver different drugs that can enhance each other’s anti-sarcoma activity by targeting multiple processes related to mitochondria. Safety and activity in patients must still be proved, but further developments of nanocarriers look promising for their advantages in terms of selectivity and efficacy compared to free drugs.

## 4. Moving Toward Clinical Applications of Mitochondria-Targeting Therapies

### 4.1. Combination Strategies with Chemotherapy and Immunotherapy

Although natural products and nanomedicines represent promising future therapeutic approaches, combining mitochondrial-targeting agents with established anticancer treatments currently represents the most clinically realistic strategy. Such combinations may enhance efficacy, overcome resistance, and potentially reduce the dose-dependent toxicity associated with conventional chemotherapy or immunotherapy.

At preclinical level, ascorbate has been shown to enhance cisplatin cytotoxicity in OS cells by increasing ROS production and inducing mitochondrial dysfunction. This effect is also appreciable in resistant OS stem cell populations characterized by elevated mitochondrial activity [81], highlighting the relevance of mitochondrial targeting in overcoming chemoresistance. The natural compound hesperidin also potentiates cisplatin efficacy by activating mitochondria-dependent apoptosis [82], while oridonin [83] and ivermectin [84] act in the same way, enhancing the efficacy of doxorubicin, potentially allowing dose reduction of anthracyclines and limiting cardiotoxicity. Currently, these strategies are limited to preclinical investigations. The key limitation for the clinical translation of most of these compounds is their potential toxicity to healthy tissues. Finding a proper therapeutic window for most compounds has still not been explored, because there is not a quantitative difference between the energetic and molecular pathways controlled by mitochondria in sarcomas and in healthy tissues. Dosing mitochondria-targeting drugs to impact sarcomas but not non-transformed tissues requires substantial amounts of preclinical work before being translated into a phase I trial. Also, drug–drug interactions between mitochondria-targeting agents and chemotherapeutic drugs currently used in sarcoma treatment, an impact on drug pharmacokinetics of such combinations, must be carefully evaluated before designing the first clinical interventions.

As anticipated, a step toward an increased selectivity toward sarcoma cells over healthy tissues can be offered by delivery technologies based on nanoformulations that grant a more selective delivery to tumors. Among the most promising strategies in the context of clinical application, there is a mitochondria-targeted nanoplatform combining doxorubicin with the photosensitizer IR780 that has been successfully tested in murine OS as near-infrared photodynamic therapy: upon irradiation, doxorubicin was significantly accumulated within mitochondria, increasing ROS and apoptosis, but also recruiting CD4^+^ and CD8^+^ T lymphocytes within the TME [85]. Implementing nanomedicine-based formulations and eventually finding active targeting strategies toward sarcomas may boost the translational efficiency of mitochondria-targeting agents into clinical settings.

Targeting mitochondrial pathways may also influence the responses to immunotherapy. For instance, metformin has been shown to improve the efficacy of immune checkpoint inhibitors by limiting mitochondrial oxygen consumption [86]: this relieves the immune suppression characterizing a hypoxic TME [87]. Interestingly, the immune checkpoint inhibitor atezolizumab induces mitochondrial dysfunction, ROS accumulation, and mitochondria-dependent apoptosis in OS cells, but once again sarcoma cells show their remarkable adaptability by activating protective mitophagy that limits the therapeutic efficacy. The concurrent use of chloroquine, a pharmacological inhibitor of autophagy/mitophagy, rescues atezolizumab-induced apoptosis and improves tumor control in preclinical models of OS [88], suggesting that co-targeting mitochondrial stress responses may potentiate immune checkpoint blockade.

The effects of mitochondrial modulation on immunotherapy are largely dependent on the heterogeneity of the TME. Most sarcomas are considered immunologically “cold”; however, the abundance and functional state of tumor-infiltrating lymphocytes (TIL), tertiary lymphoid structures, cancer-associated fibroblasts, and tumor-associated macrophages vary substantially among and within histotypes [89]. M2-like macrophages and cancer-associated fibroblasts can promote immune suppression and T-cell exclusion, whereas high densities of CD8^+^ lymphocytes, PD-L1-expressing immune cells, and B-cell-rich tertiary lymphoid structures have been associated with greater sensitivity to immune checkpoint inhibitors. Consistently, responses to PD-1/PD-L1 blockade have been more frequently observed in selected immune-enriched subtypes of sarcomas, including undifferentiated pleomorphic sarcoma, dedifferentiated LS, and alveolar STS, while limited activity has been reported in several other histotypes [89]. Mitochondrial metabolism may contribute to this variability by regulating tumor oxygen consumption, ROS production, and the release of immunogenic DAMP. High tumor-cell OXPHOS may aggravate hypoxia by depriving the TME of oxygen and impair cytotoxic immune-cell activity. At the same time, excessive mitochondrial ROS and mitochondrial damage can promote immunogenic cell death, antigen presentation, recruitment of CD8^+^ T cells and pro-inflammatory macrophages [74,75,77,85]. Conversely, protective mitophagy limits these immunogenic signals and reduces the efficacy of immune checkpoint blockade, as suggested by the enhanced activity of atezolizumab combined with chloroquine in OS models [88]. Nevertheless, mitochondrial fitness is also required for lymphocyte survival and effector activity; consequently, non-selective mitochondrial inhibition could suppress antitumor immunity. Future combination strategies should therefore evaluate mitochondrial effects separately in tumor and immune cells and incorporate histotype-specific TME biomarkers, including TIL, macrophage polarization, and PD-L1 expression, to identify patients most likely to benefit from mitochondrial-targeting agents combined with immunotherapy.

### 4.2. Clinical Translation of Mitochondrial-Targeting Approaches

Despite the promising results on combination regimens incorporating mitochondria-targeting strategies in sarcoma treatment at a preclinical level, the clinical evaluation of such therapies remains limited. Currently, most ongoing studies are early-phase, non-randomized clinical trials designed primarily to assess the safety and feasibility of repurposed drugs fulfiling mitochondrial functions.

For instance, the combination of disulfiram/copper with liposomal doxorubicin in refractory sarcomas (NCT05210374) or with standard treatments in OS (NCT07477457) and the combination of metformin with current systemic therapies in advanced STS (NCT07291297) and with maintenance therapy in high-risk OS and ES patients (NCT04758000) are under evaluation as adjuvant approaches to chemotherapy and radiotherapy. Hydroxychloroquine is currently being investigated in combination with gemcitabine and docetaxel for recurrent OS (NCT03598595), based on previous studies showing that hydroxychloroquine with sirolimus induces metabolic reprogramming in STS, despite limited clinical efficacy [90]. Among the completed studies, high-dose ascorbate has shown acceptable safety profiles but limited efficacy when combined with radiotherapy or gemcitabine-based regimens in advanced STS and OS (NCT03508726; NCT03468075). Nevertheless, evidence of immune activation and favorable tolerability suggests that further investigations are warranted in selected patient populations.

TCA-cycle-targeting agents have also been tested in clinical trials, taking advantage of the development of *IDH1* inhibitors widely developed in solid and hematological tumors: AG-120/Ivosidenib was the first drug tested in a phase I trial designed for CS (NCT06127407), showing long-term control of disease and acceptable toxicity [20]. Other *IDH1* inhibitors are under evaluation in phase I trials on solid tumors characterized by *IDH1* mutations, including sarcomas: FT-2102 (NCT03684811), IDH305 (NCT02381886), BAY1436032 (NCT02746081), and AG-881 (NCT02481154). Only one phase II trial is investigating the use of the *IDH1* inhibitor AG-120/Ivosidenib in combination with Nivolumab as a strategy superior to immunotherapy alone in *IDH1*-mutant tumors (NCT04056910). Except for the NCT06127407 study, the other trials are not limited to sarcomas: given the low frequency of sarcomas compared to other solid tumors and the heterogeneity of sarcomas, a huge number of patients must be enrolled to reach statistical power before drawing conclusions on the efficacy of *IDH1* inhibitors in specific histotypes and subsets of sarcomas.

Overall, most clinical studies have been focused on drugs or supplements already used for other clinical applications or other tumors, whose pharmacokinetic profile and side effects are well known. For other agents targeting mitochondrial metabolism, redox homeostasis, and autophagy, the clinical applications are preliminary, mainly because of the lack of safe therapeutic windows identified in preclinical studies. Understanding how mitochondria-targeting agents can be optimally integrated with the current standards of care may help us to select agents characterized by good efficacy and safety and that are worth further development in phase I clinical trials.

An integrated overview of the therapeutic strategies discussed above is presented in Table 2, summarizing the main agents and formulations that directly target mitochondria, downstream mitochondria-dependent pathways or upstream pathways controlling mitochondrial biology. For each approach, specific mitochondrial target or process, the mechanism of action, sarcoma subtype, experimental model, therapeutic effect, level of evidence, and current clinical status are reported.

## 5. Conclusions and Future Perspectives

Accumulating evidence indicates that mitochondria are far from being simple energy-producing organelles in sarcomas. Alterations in mitochondrial metabolism, dynamics, redox homeostasis, and mitochondria-dependent apoptosis actively contribute to malignant transformation, tumor progression, metastatic dissemination, immune evasion, and resistance to chemotherapy. The studies discussed in this review demonstrate that sarcoma cells exploit mitochondrial plasticity to adapt to environmental and therapeutic pressures, while also revealing several mitochondrial processes including OXPHOS, mitochondrial transport systems, redox-regulating networks, and mitochondrial dynamics as promising therapeutic vulnerabilities.

Despite these advances, the clinical translation of mitochondria-targeted therapies remains challenging. Most evidence derives from preclinical studies, whereas clinical trials evaluating mitochondria-targeting agents in patients remain limited. This is partly attributable to the rarity and marked heterogeneity of sarcomas that make patient recruitment more complicated and hinder the development of universally effective therapeutic approaches. Furthermore, the safety, specificity, and long-term effects of drugs targeting mitochondrial functions require careful evaluation, given the essential role of mitochondria in normal tissues.

Future research should therefore move toward a precision medicine approach based on the mitochondrial phenotype of individual sarcomas. Since sarcomas are a group of highly heterogeneous tumors, also characterized by high intratumor heterogeneity, establishing mitochondria-targeted precision medicine, although appealing in theory, may encounter some limitations in practice. In GIST with documented loss of SDHB or in CS with oncogenic mutations of IDH1, the use of TCA cycle inhibitors designed to prevent the accumulation of oncometabolites—such as IDH1 inhibitors—can be considered the first examples of mitochondria-oriented precision medicine. Comprehensive molecular and metabolic profiling, including functional metabolic assays and metabolomic profiles—feasible on patient-derived organoids, bioptic or surgery samples, respectively—may enable the identification of patient subsets that are particularly dependent on specific mitochondrial pathways and could be ideal candidates to benefit from targeted interventions. Performing such multi-omic analysis in sarcomas is still at the beginning but it is the most straightforward way to build mitochondria-targeting precision treatments in the future.

Importantly, bone sarcomas offer unique opportunities for the development of innovative drug-delivery strategies. The distinctive biological properties of bone tissue may facilitate the selective delivery of mitochondria-targeted agents through bone-seeking platforms, including calcium phosphate nanoparticles, amino bisphosphonate-based conjugates, and other biomaterials with high bone affinity. Such approaches could improve drug accumulation within the TME while minimizing systemic toxicity. The integration of mitochondrial biology with precision oncology and advanced drug-delivery technologies may pave the way for more effective therapeutic strategies and improved outcomes for patients with bone and soft-tissue sarcomas.

## Figures and Tables

**Figure 1:**
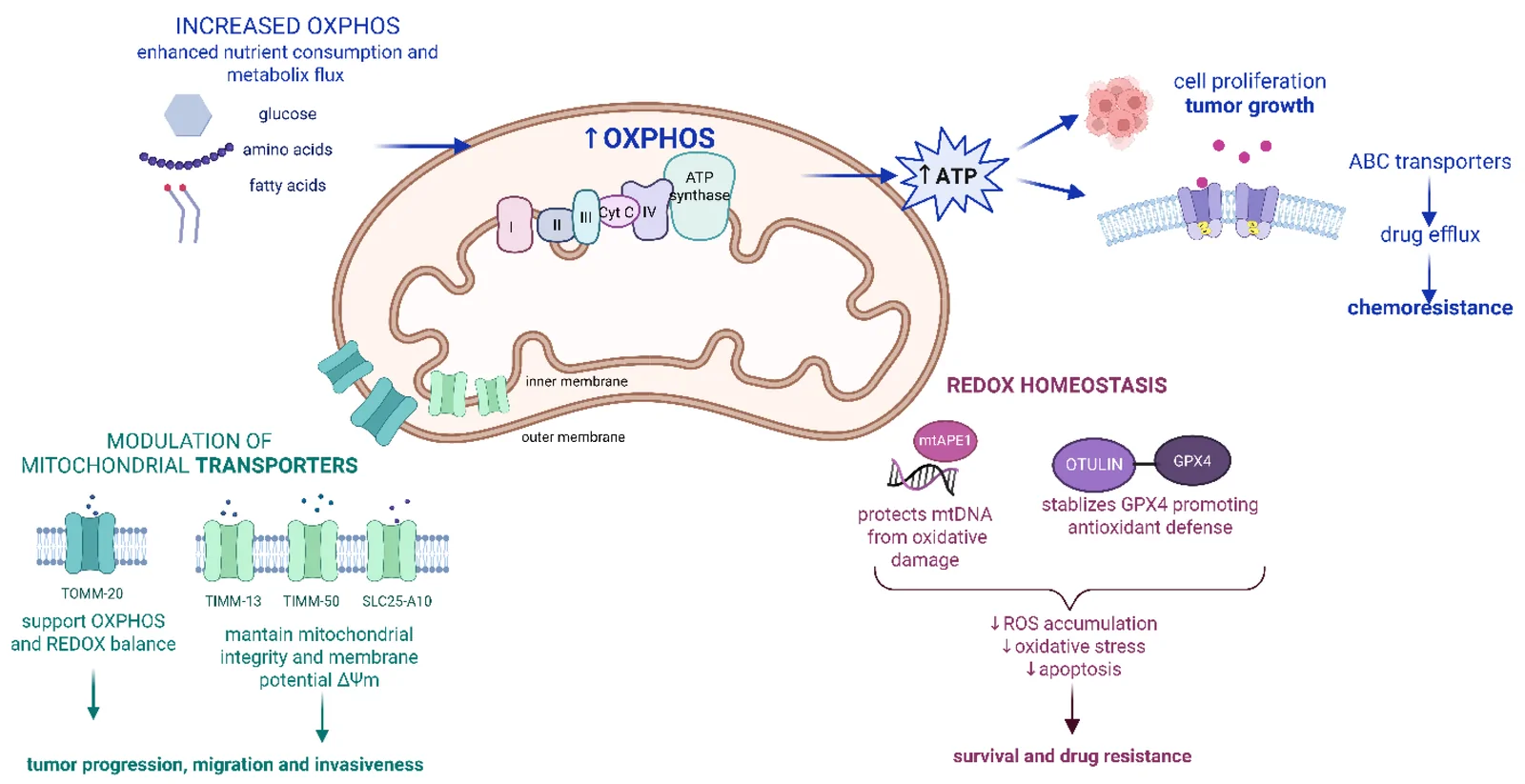
Alterations in mitochondria energetic metabolism in sarcoma. Increased oxidative phosphorylation, alteration of mitochondrial transporters and oxidoreductive (REDOX) metabolism promote increased ATP, which fuels drug efflux via ABC transporters, and decreased ROS, relieving OS cells from pro-apoptotic signals. These events contribute to cell survival and drug resistance. Created in BioRender. Riganti, C. (2026) https://BioRender.com/b3zx8u7.

**Figure 2:**
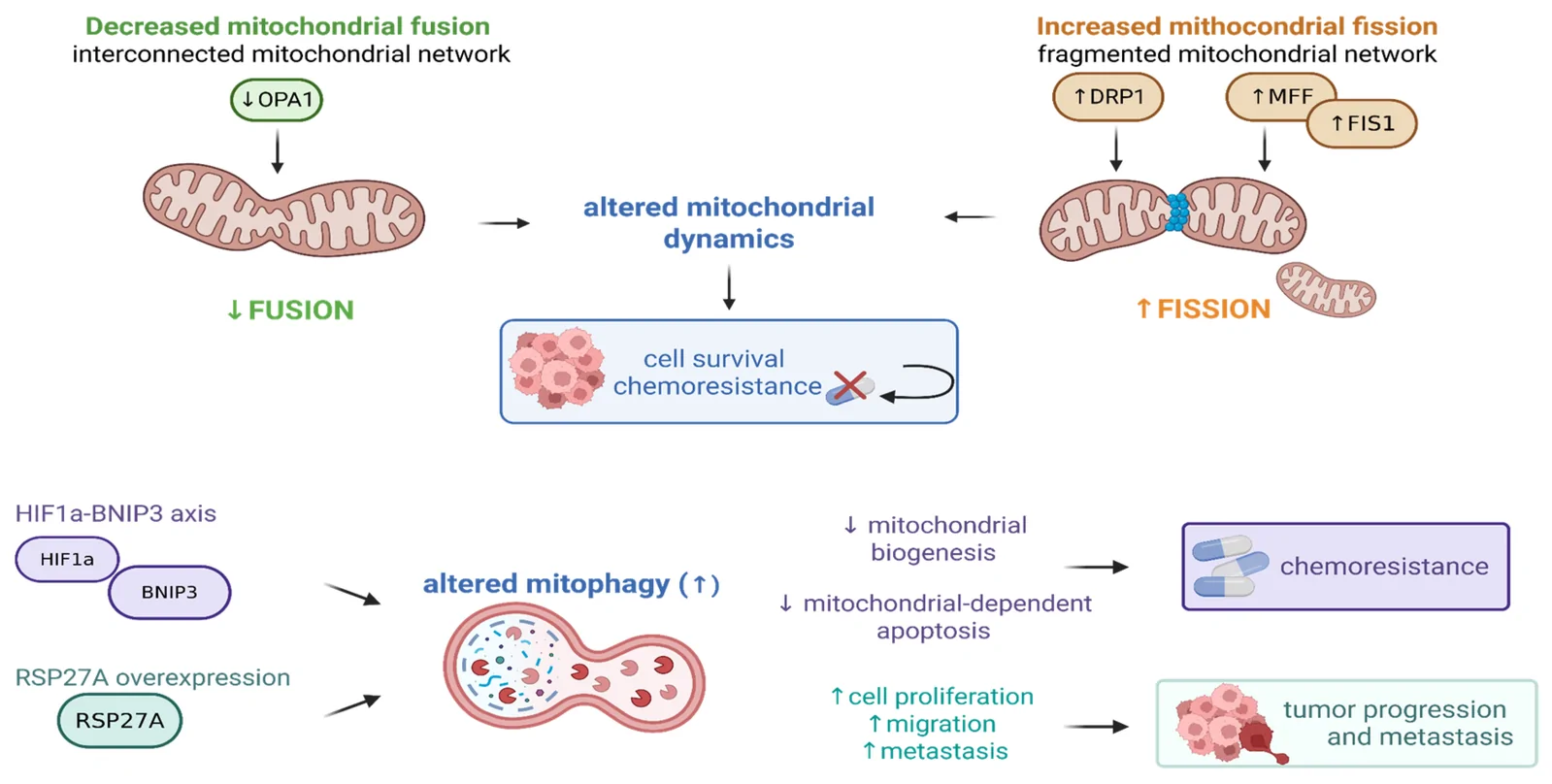
Alterations in mitochondria dynamics in sarcoma. Decreased fusion, increased fission and altered autophagy reduce mitochondrial biogenesis and production of ATP. As a result, sarcoma cells are more aggressive, more likely to metastasize and more chemoresistant. Created in BioRender. Riganti, C. (2026) https://BioRender.com/so8n0w3.

**Figure 3:**
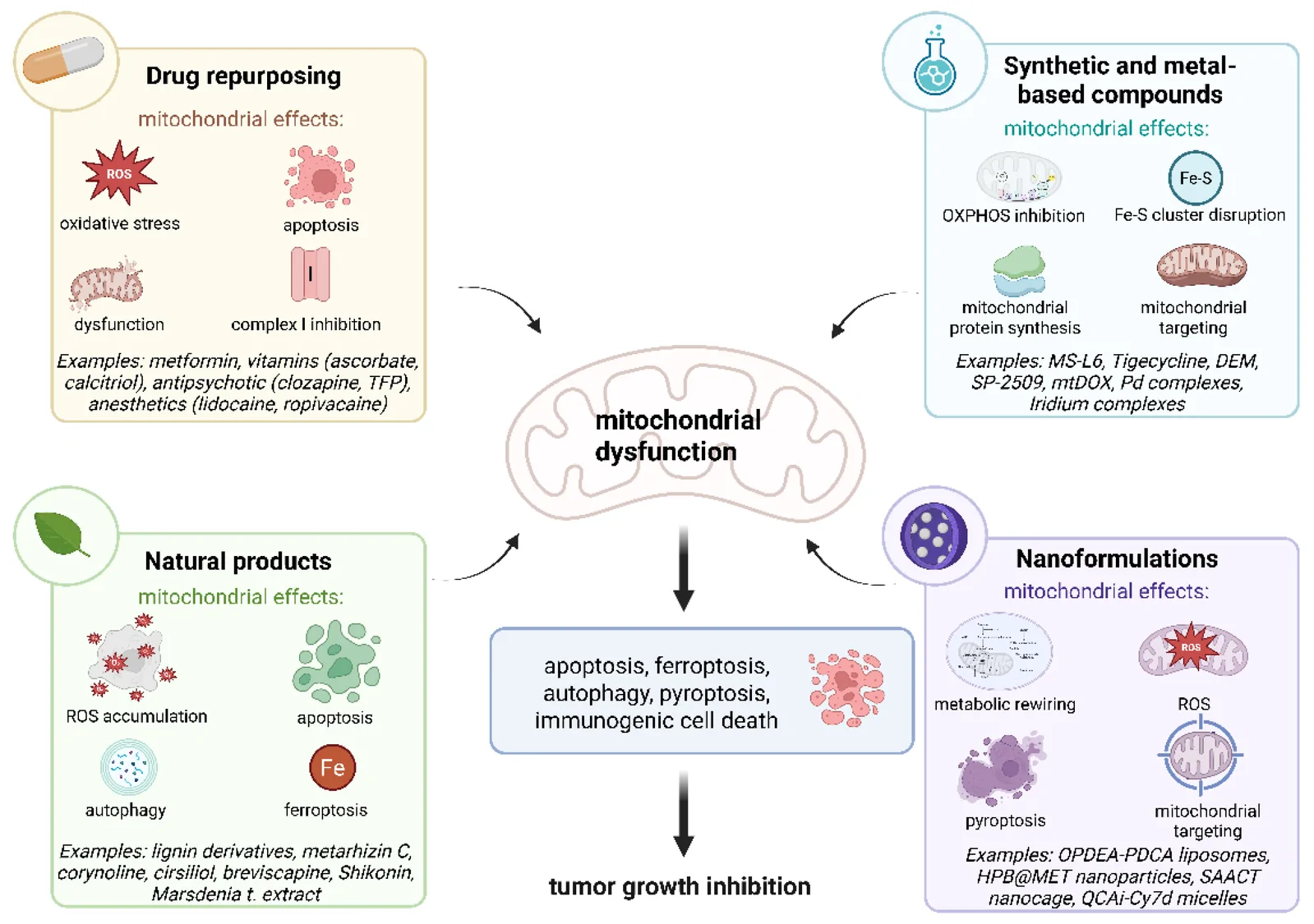
Overview of different strategies targeting mitochondria in sarcomas. Created in BioRender. Riganti, C. (2026) https://BioRender.com/t5a53co.

**Table 1:** Mechanisms of drug resistance induced by mitochondria-related process in sarcomas.

Biological Process	Mechanisms	Sarcoma Type	Experimental Model	Resistance Type	References
Deregulated TCA cycle	SDH inhibition by TGF-β, accumulation of succinate and activation of HIF-1α-dependent pathways	OS	In vitro and in vivo	Resistance to cisplatin and methotrexate	[18]
High energetic metabolism	Increased OXPHOS and production of ATP that favors the catalytic efficiency of ABC transporters	OS	In vitro	Resistance to doxorubicin	[30]
Loss of genes encoding for complex III and IV	Loss of activation of LSD-1 inhibitors by complex III and IV	ES	In vitro	Resistance to LSD-1 inhibitor SP-2509	[31]
Increased expression of ANT3	EFHD1-ANT3-mediated activity of OXPHOS and mitochondrial ROS buffering	OS	In vitro and in vivo	Resistance to cisplatin	[26]
Increased mitobiogenesis	RORγ-mediated activation of PGC1β, increased mitobiogenesis, FAO- and OXPHOS-dependent production of ATP	OS	In vitro and in vivo	Resistance to cisplatin and methotrexate	[32]
Increased import of mitochondrial proteins	Increased expression of TOMM20	CS	In vitro	Resistance to doxorubicin, gemcitabine and palbociclib	[33]
Increased fission/fusion ratio	Increased expression of DRP1 protein, larger mitochondrial network, higher OXPHOS activity	OS	In vitro	Resistance to cisplatin and doxorubicin	[41]
	Increased expression of MFF/FIS1, ampler mitochondrial network, higher OXPHOS activity	RMS	In vitro	Resistance to cisplatin, doxorubicin, topotecan	[42]

ABC: ATP-binding cassette transporters; ANT3: adenine nucleotide translocator 3; CS: chondrosarcoma; DRP1: dynamin-related protein 1; EFHD1: EF-hand domain-containing protein 1; ES: Ewing sarcoma; FAO: fatty acid oxidation; FIS1: mitochondrial fission 1 protein; HIF-1α: hypoxia-inducible factor 1 alpha; LSD-1: lysine-specific demethylase 1; MFF: mitochondrial fission factor; OS: osteosarcoma; OXPHOS: oxidative phosphorylation; PGC1β: peroxisome proliferator-activated receptor gamma coactivator 1 beta; RMS: rhabdomyosarcoma; RORγ: retinoic acid receptor-related orphan receptor gamma; ROS: reactive oxygen species; SDH: succinate dehydrogenase; TCA: tricarboxylic acid cycle; TGF-β: transforming growth factor beta; TOMM20: translocase of outer mitochondrial membrane 20.

**Table 2:** Integrated summary of mitochondria-targeting agents in sarcoma: targets, therapeutic strategies, mechanisms and effects, experimental evidence, clinical status.

Mitochondrial Target/Pathway	Therapeutic Agent/Strategy	Sarcoma Subtype	Mechanism and Therapeutic Effect	Experimental Model and Level of Evidence	Current Clinical Status
EFHD1-ANT3 axis/mPTP	Carboxyatractyloside + cisplatin	OS	Mechanism: ANT3 inhibition, mPTP opening Effect: mitochondrial fitness alteration, cisplatin sensitization [26]	Model: cisplatin-resistant cells; in vivo model Evidence: in vitro and in vivo	Not clinically evaluated in sarcoma
OXPHOS/P-gp-mediated efflux	mtDox	OS	Mechanism: mitochondria localization, P-gp efflux bypass Effect: doxorubicin sensitization [30]	Model: doxorubicin-resistant human OS cells Evidence: in vitro and in vivo	Not clinically evaluated
*MTAP*-C1orf112/TCA-OXPHOS	Methionine deprivation	OS	Mechanism: C1orf112 reduced activation, reduced TCA cycle, OXPHOS and ROS buffering Effect: reduced tumor growth, increased apoptosis [29]	Model: cell lines; PDX Evidence: in vitro and in vivo (PDX)	Not clinically evaluated in sarcoma
RORɣ-PGC1β/FAO and mitochondrial biogenesis	RORɣ inhibition	OS	Mechanism: PGC1β decreased, lower OXPHOS and FAO Effect: Reduced tumor growth, increased apoptosis, decreased transporter-mediated resistance [12]	Model: cell lines; methotrexate- and cisplatin-resistant PDX Evidence: in vitro and in vivo (PDX)	Not clinically evaluated in sarcoma
SDH-HIF1*α* metabolic switch	HIF1*α* inhibitor + methotrexate or cisplatin	OS	Mechanism: reversion of TGFβ-induced glycolytic shift caused by SDH suppression and HIF1*α* activation Effect: chemosensitization [18]	Model: chemotherapy-treated cell and in vivo models Evidence: in vitro and in vivo	Not clinically evaluated in sarcoma
*SOX6*-*TXNIP*/mitochondrial redox balance	Elesclomol	ES	Mechanism: increased mitochondrial ROS beyond the cytotoxic threshold. Effect: cell death, chemosensitization [40]	Model: *SOX6*-high ES models Evidence: preclinical	Not clinically evaluated in ES
ROS-Ca^2+^ signaling/OXPHOS	High-dose ascorbate	OS; advanced STS	Mechanism: increased mitochondrial ROS, disrupted Ca^2+^ homeostasis, membrane potential and OXPHOS, decreased ATP. Effect: Cell death, reduced tumor growth, cisplatin sensitization [52,81]	Model: OS cells and stem-like cells; xenografts; patients Evidence: in vitro and in vivo and clinical	NCT03508726 and NCT03468075 completed: acceptable tolerability but limited efficacy
Mitochondrial architecture/intrinsic apoptosis	Calcitriol	RMS	Mechanism: disrupted mitochondrial architecture, increased mitochondria-dependent apoptosis Effect: cell-cycle arrest, apoptosis [53]	Model: RMS cell lines Evidence: in vitro	Approved vitamin D analog; not clinically evaluated for RMS
Mitochondrial redox homeostasis/ fission	Clozapine	Liposarcoma	Mechanism: Increased mitochondrial oxidative stress and fragmentation. Effect: early mitochondrial dysfunction, cell death [54]	Model: SW872 human liposarcoma cells Evidence: in vitro	Approved antipsychotic; not clinically evaluated for liposarcoma
AKT-TXNIP/mitochondrial ROS	Trifluoperazine	OS	Mechanism: AKT inhibited, TXNIP, mitochondrial ROS and depolarization increased Effect: reduced tumor growth, increased apoptosis [55]	Model: cell lines; xenografts Evidence: in vitro and in vivo	Approved antipsychotic; not clinically evaluated for OS
Complex I/mitochondrial bioenergetics	Metformin	OS; advanced STS; high-risk OS/ES	Mechanism: complex I inhibited, decreased ATP, increased ROS. Effect: reduced tumor growth/metastatic progression, increased apoptosis [56]	Model: OS stem-like cells; xenografts; clinical protocols Evidence: in vitro, in vivo and clinical investigation	Trials registered: NCT07291297 (advanced STS) and NCT04758000 (OS/ES maintenance)
Complex I and HIF1*α* signaling	Metformin + nitroglycerin	FS	Mechanism: impaired OXPHOS and HIF1*α*a-dependent signaling. Effect: Reduced tumor growth and vascularization, increased apoptosis [57]	Model: Hamster fibrosarcoma model Evidence: in vivo	Not clinically evaluated in sarcoma
Mitochondrial redox stress/intrinsic apoptosis	DSF-Cu	OS; refractory sarcomas	Mechanism: increased ROS, mitochondrial depolarization, cytochrome c release. Effect: reduced tumor growth, increased apoptosis [47]	Model: OS cell and in vivo models; clinical protocols Evidence: in vitro and in vivo and clinical investigation	Trials registered: NCT05210374 (refractory sarcomas) and NCT07477457 (OS)
Mitochondrial dynamics and membrane potential	Lidocaine or ropivacaine	OS	Mechanism: increased mitochondrial fragmentation, depolarization and ROS accumulation. Effect: reduced proliferation, migration and invasion, increased apoptosis [59]	Model: SaOS-2 cells Evidence: in vitro	Approved local anesthetics; not clinically evaluated for OS
ETC complex I/uncoupling	MS-L6	RMS	Mechanism: complex I inhibited, mitochondrial uncoupling, ATP depletion. Effect: cytostatic and cytotoxic effects with limited systemic toxicity [60]	Model: RMS cells; in vivo model Evidence: in vitro and in vivo	Not clinically evaluated
Fe-S proteins in ETC complexes I and III	SP-2509	ES	Mechanism: Fe-S-dependent respiratory proteins destabilized, independently of LSD1 inhibition. Effect: reduced respiratory capacity and viability [62]	Model: ES cells Evidence: in vitro	Not clinically evaluated for this mechanism
Mitochondrial membrane potential/cytochrome c	Palladium(II) oxime complexes	OS	Mechanism: lysosomal and mitochondrial depolarization, cytochrome c release Effect: stronger apoptosis and antitumor activity than cisplatin [63]	Model: cell lines; in vivo comparative model Evidence: in vitro and in vivo	Not clinically evaluated
mPTP/mitochondrial ROS	Photoactivatable iridium(III) complex	RMS	Mechanism: Accumulation in mitochondria after light irraditaion, increased ROS and mPTP opening Effect: increased necrotic death, apoptosis in resistant cells [64]	Model: RMS cells; cancer stem cell-enriched populations Evidence: in vitro	Not clinically evaluated
ETC proteins/mitochondrial redox balance	Phenethyl isothiocyanate (PEITC)	OS	Mechanism: increased mitochondrial ROS, depleted respiratory-chain proteins and ATP, depolarized mitochondria Effect: Increased apoptosis, ferroptosis and autophagy [65]	Model: OS cell models Evidence: in vitro	Not clinically evaluated
Mitochondrial ribosomes/ETC translation	Tigecycline	OS	Mechanism: translation of mitochondrially encoded respiratory-chain proteins inhibited Effect: reduced tumor growth [66]	Model: Cells with high mitochondrial biogenesis; in vivo models Evidence: in vitro and in vivo	Approved antibiotic; not clinically evaluated for OS
*METTL17*/ mitochondrial translation and OXPHOS	Demethylzeylasteral (DEM)	OS	Mechanism: inhibited *METTL17*-dependent mitochondrial RNA modification and mitoribosome assembly, reduced ETC proteins and ATP, depolarized mitochondria. Effect: reduced tumor growth [24]	Model: Cell lines; xenografts Evidence: in vitro and in vivo	Not clinically evaluated
Glutathione-redox balance/mPTP	Modified lignin derivatives	OS	Mechanism: depleted glutathione, increased ROS, mPTP opening and cytochrome c release Effect: increased apoptosis [67]	Model: OS cell models Evidence: in vitro	Not clinically evaluated
Mitochondrial respiration	Metarhizin C	OS	Mechanism: selectively inhibited mitochondrial respiration, particularly during glucose deprivation. Effect: preferential death of OXPHOS-dependent cells [68]	Model: OS cell models under glucose-replete/deprived conditions Evidence: in vitro	Not clinically evaluated
Src-JNK/intrinsic apoptosis	Corynoline	OS	Mechanism: Activated Src/JNK signaling, mitochondrial damage and membrane-potential loss. Effect: reduced tumor growth, increased apoptosis [69]	Model: cell lines; in vivo Evidence: in vitro and in vivo	Not clinically evaluated
PI3K-AKT-*FOXO1*/apoptosis and autophagy	Cirsiliol + methotrexate	OS	Mechanism: inhibited PI3K/AKT axis, activated *FOXO1*, increased mitochondrial apoptosis/autophagy Effect: methotrexate sensitization [70]	Model: methotrexate-resistant OS models Evidence: in vitro and in vivo	Not clinically evaluated
*Bax*-cytochrome c-caspase pathway	Breviscapine	OS	Mechanism: increases *Bax*, cytochrome c release and caspase activation Effect: reduced tumor growth, increased apoptosis [71]	Model: SaOS-2 cells; in vivo model Evidence: in vitro and in vivo	Not clinically evaluated
Mitochondrial ROS-HIF1*α*-HO-1 axis	Shikonin	OS	Mechanism: increases mitochondrial ROS and activates the HIF1*α*/HO-1 axis. Effect: induces ferroptosis [72]	Model: OS cell models Evidence: in vitro	Not clinically evaluated
Iron/ROS-*PINK1*-Parkin mitophagy	*Marsdenia tenacissima* extract	OS	Mechanism: iron accumulation, oxidative stress and *PINK1*/Parkin-dependent mitophagy Effect: reduced tumor growth, increased ferroptosis [73]	Model: cell lines; in vivo model Evidence: in vitro and in vivo	Not clinically evaluated
*PDK1*/glycolysis-OXPHOS shift	Mitochondria-targeted OPDEA-PDCA micelles	OS	Mechanism: dichloroacetate delivery to mitochondria, inhibited *PDK1*, increase OXPHOS-derived stress. Effect: reduced tumor growth, increased pyroptotic/immunogenic cell death [74]	Model: cell lines; xenografts Evidence: in vitro and in vivo	Not clinically evaluated; nanomedicine remains preclinical
Mitochondrial Ca^2+^ overload/copper-amplified ROS	pH-sensitive Ca^2+^/Cu_2_O/elesclomol nanocages	OS	Mechanism: increased TCA/OXPHOS activity, Cu-amplified mitochondrial ROS burst. Effect: increased immunogenic cell death, activated activates CD8^+^ T cells and M1 macrophages [77]	Model: cell lines; murine tumor models Evidence: in vitro and in vivo	Not clinically evaluated; nanomedicine remains preclinical
Mitochondrial ROS	Prussian Blue + metformin mesoporous silica nanoparticles	OS	Mechanism: H_2_O_2_-to-hydroxyl-radical conversion, damaged mitochondria. Effect: amplified oxidative damage, reduced tumor growth [78]	Model: cell lines; xenografts Evidence: in vitro and in vivo	Not clinically evaluated; nanomedicine remains preclinical
*PIM1*-DRP1/mitochondrial dynamics	QCAi-Cy7d	OS	Mechanism: *PIM1* inhibitor delivery to mitochondria, DRP1 activity inhibited Effect: reduced tumor growth, increased apoptosis [79]	Model: molecular docking; cell models; xenografts Evidence: in vitro and in vivo	Not clinically evaluated; theranostic nanomedicine remains preclinical
Mitochondrial ROS/intrinsic apoptosis	Hesperidin + cisplatin	OS	Mechanism: enhances cisplatin-induced mitochondrial apoptotic signaling. Effect: inhibits proliferation and potentiates apoptosis [82]	Model: human OS cells Evidence: in vitro	Not clinically evaluated
Mitochondrial apoptosis	Oridonin + doxorubicin	OS	Mechanism: enhanced doxorubicin-induced mitochondrial apoptosis Effect: doxorubicin sensitization [83]	Model: human OS cells Evidence: in vitro	Not clinically evaluated
Mitochondrial dysfunction/apoptosis	Ivermectin + chemotherapy	OS	Mechanism: enhanced doxorubicin-induced mitochondrial dysfunction and apoptosis. Effect: chemosensitization [84]	Model: OS models Evidence: Preclinical	Approved antiparasitic; not clinically evaluated for OS
Mitochondrial ROS/photodynamic injury	Doxorubicin-IR780 biomimetic nanoplatform + near-infrared light	OS	Mechanism: doxorubicin and photosensitizer codelivery to mitochondria, increased ROS after irradiation. Effect: increases apoptosis and CD4^+^/CD8^+^ T cell recruitment [85]	Model: cell models; murine OS Evidence: in vitro and in vivo	Not clinically evaluated; nanomedicine remains preclinical
Mitochondrial ROS and protective autophagy	Atezolizumab + chloroquine	OS	Mechanism: blocked protective autophagy induced by checkpoint-inhibitor and mitochondrial ROS. Effect: increased tumor control and apoptosis [88]	Model: OS cells; in vivo model Evidence: in vitro and in vivo	Combination not clinically evaluated in OS
Autophagy/mitochondrial metabolism	Hydroxychloroquine + gemcitabine/docetaxel or sirolimus	Recurrent OS; STS	Mechanism: blocked lysosomal autophagic flux and stress adaptation. Effect: metabolic reprogramming combined with sirolimus [90]	Model: patients in early-phase, non-randomized studies Evidence: clinical	NCT03598595 registered for recurrent OS; prior STS study completed

ES, Ewing sarcoma; ETC, electron transport chain; FAO, fatty acid oxidation; FS, fibrosarcoma; mPTP, mitochondrial permeability transition pore; OS, osteosarcoma; OXPHOS, oxidative phosphorylation; PDX, patient-derived xenograft; RMS, rhabdomyosarcoma; ROS, reactive oxygen species; STS, soft-tissue sarcoma.

## Data Availability

No new data were created or analyzed in this study. Data sharing is not applicable to this article.

## Abbreviations

The following abbreviations are used in this manuscript:

ABC	ATP Binding Cassette
ACLY	ATP-Citrate Lyase
ACOT9	Acyl-Coa Thioesterase 9
AKT	Protein Kinase B
ANT	Adenine Nucleotide Translocator
ARNT2	Aryl hydrocarbon receptor nuclear translocator 2
ASS1	Argininosuccinate Synthetase 1
ATG4D	Autophagy-related protein 4D
ATP6V1E1	Atpase H+ Transporting V1 Subunit E1
BAX	BCL2-associated X protein
BNIP3	BCL2 Interacting Protein 3
BNIP3L	BNIP3 like
C-MYC	Cellular Myelocytomatosis
COX	Cytochrome C Oxidase
CPT1	Carnitine Palmitoyltransferase 1
CS	Chondrosarcoma
DAMP	Damage-Associated Molecular Pattern
DCA	Dichloroacetate
DEM	Demethylzeylasteral
DRP1	Dynamin-Related Protein 1
DSF-Cu	Disulfiram combined with Copper
ECAR	Extracellular Acidification Rate
EFHD1	EF-Hand Domain-Containing Protein 1
ES	Ewing Sarcoma
ESBR1-FLI1	Ewing sarcoma breakpoint region 1–Friend leukemia integration 1
ETC	Electron Transport Chain
FAHD1	Fumarylacetoacetate Hydrolase Domain-Containing 1
FAO	Fatty Acid Oxidation
FASN	Fatty Acid Synthase
FDX1	Ferredoxin 1
FIS1	Fission 1 Protein
FOXO1	Forkhead box protein O1
FS	fibrosarcoma
FUNDC1	FUN14 Domain-Containing 1
GABARAP	GABA type A Receptor-Associated Protein
GLS	Glutaminase
GIST	Gastrointestinal stromal tumor
GLUT1	Glucose Transporter 1
GPX4	Glutathione Peroxidase 4
HES5	Hes Family bHLH Transcription Factor 5
HIF1α	Hypoxia-Inducible Factor-1α
HK2	Hexokinase 2
HMGB1	High-Mobility Group Box 1
HMGCR	HMG-Coa Reductase
HO-1	Hemeoxygenase-1
HS3ST4	Heparan Sulfate-Glucosamine 3-Sulfotransferase 4
IDH1	Isocitrate dehydrogenase 1
INFɣ	Interferon gamma
JNK	c-Jun N-terminal kinase
KLK2	Kallikrein-Related Peptidase 2
KRAS	Kirsten rat sarcoma virus oncogene homolog
LDHA	Lactate Dehydrogenase A
LS	Liposarcoma
LSD1	Lysine-Specific Demethylase-1
METTL17	Methyltransferase-Like 17
MFF	Mitochondrial Fission Factor
MFN2	Mitofusin 2
mPTP	Mitochondrial Permeability Transition Pore
MRI	Magnetic Resonance Imaging
MSC	Mesenchymal Stem Cells
MTAP	Methylthioadenosine Phosphorylase
mtAPE1	Mitochondrial APE1
MT-CO2/3	Cytochrome c oxidase subunit 2/3
mtDox	mitochondrial-targeted doxorubicin
MT-ND1	Mitochondrial-NADH dehydrogenase subunit 1
NDUFB9	NADH:Ubiquinone Oxidoreductase Subunit B9
NIX	NIP-3-like protein X
NRXN1	Neurexin 1
OCR	Oxygen Consumption Rate
OPDEA	Poly[2-(N-oxide-N,N-diethylamino)ethyl methacrylate]
OR2W3	Olfactory Receptor Family 2 Subfamily W Member 3
OS	Osteosarcoma
OXPHOS	Oxidative Phosphorylation
PARKIN	PARKIN RBR E3 Ubiquitin Protein Ligase
PDK1	Pyruvate Dehydrogenase Kinase 1
PD-L1	Programmed Death-Ligand 1
PEITC	Phenethyl isothiocyanate
PFKFB	6-phosphofructo-2-kinase/fructose-2,6-bisphosphatase
PGC1β	Proliferator-Activated Receptor Gamma Coactivator 1-Β
PGM1	Phosphoglycerate Mutase 1
P-gp	P-glycoprotein
PHGDH	Phosphoglycerate Dehydrogenase
PI3K	Phosphoinositide 3-kinase
PIM1	Proto-oncogene serine/threonine-protein kinase
PINK1	PTEN-Induced Kinase 1
REDOX	reduction–oxidation
RMS	Rhabdomyosarcoma
RORγ	RAR-Related Orphan Receptor γ
ROS	reactive oxygen species
RPS27A	Ribosomal Protein S27A
SDH	Succinate Dehydrogenase
SHP2	Src homology region 2 domain-containing phosphatase-2
SLC	Solute Carrier Family
SOX6	SRY-box transcription factor 6
SRC	Spare Respiratory Capacity
STS	Soft-Tissue Sarcoma
TCA	Tricarboxylic Acid
TFP	Trifluoperazine
TP53INP2	Tumor protein p53-inducible nuclear protein 2
TPP	Triphenylphosphonium
TGFβ	Transforming Growth Factor-β
TIL	Tumor-infiltrating lymphocyte
TIMM	Translocase Of The Inner Mitochondrial Membrane
TME	Tumor Microenvironment
TOMM	Translocase Of The Outer Mitochondrial Membrane
TrxR	Thioredoxin reductase
TXNIP	Thioredoxin-Interacting Protein
UQCRB	Ubiquinol-cytochrome c reductase binding protein
YAP	Yes-associated protein
